# Blood Immunopathology of Tuberculosis Patients Disrupts Monocyte‐Dependent T‐Cell Activation and Cytokine Expression

**DOI:** 10.1111/imm.70131

**Published:** 2026-03-08

**Authors:** Joseph F. Arthur, Hubert S. Ahor, Monika M. Vivekanandan, Difery Minadzi, Augustine Yeboah, Millicent Lamptey, Victoria A. Ofori, Albert D. Kegya, Rejoice Arthur, Linda A. Amoakoa, Ernest Adankwah, Dorcas O. Owusu, Mohammed K. Abass, Fredrick Gyamfi Apraku, Nana K. Ayisi‐Boateng, Seth Adane, Salisu Zakaria, Ertan Mayatepek, Julia Seyfarth, Richard O. Phillips, Marc Jacobsen

**Affiliations:** ^1^ Department of General Pediatrics, Neonatology and Pediatric Cardiology, Medical Faculty University Hospital Duesseldorf, Heinrich‐Heine University Duesseldorf Germany; ^2^ Kumasi Centre for Collaborative Research in Tropical Medicine (KCCR) Kumasi Ghana; ^3^ Agogo Presbyterian Hospital Agogo Ghana; ^4^ Kumasi South Government Hospital Atonsu Ghana; ^5^ KNUST Hospital Kumasi Ghana; ^6^ Atebubu‐Amantin Municipal Hospital Atebubu Ghana; ^7^ Sene West Health Directorate Kwame Danso Ghana; ^8^ School of Medicine and Dentistry, College of Health Sciences Kwame Nkrumah University of Science and Technology (KNUST) Kumasi Ghana

**Keywords:** impaired T‐cell response, monocytes, phytohemagglutinin, tuberculosis serum pathology

## Abstract

Pulmonary tuberculosis in humans is characterised by features of immunopathology, which influence both antimycobacterial therapy and the long‐term prognosis. In the blood of tuberculosis patients, immunopathology manifests itself in reduced immune responses to mitogenic substances. Previous studies have demonstrated the influence of tuberculosis serum on T‐cell and monocyte function, but the exact mechanisms remain unclear. Here, we performed a case/control study to analyse the influence of tuberculosis serum milieu changes on (i) T‐cell stimulation (using Staphylococcal Enterotoxin B), (ii) monocyte stimulation (using the Toll‐like receptor agonist Pam3CSK4), (iii) T‐cell/monocyte interaction characterised by the response against the lectin phytohemagglutinin, by using a novel peripheral blood mononuclear cell in vitro assay. Cell‐specific activation marker and cytokine expression were determined by multicolor flow cytometry. Staphylococcal Enterotoxin B mainly induced cytokine expression by T cells, while Pam3CSK4 stimulated monocytes to secrete distinct cytokine signatures. Phytohemagglutinin induced activation and cytokine expression in both T cells and monocytes. Notably, tuberculosis patient serum samples affected exclusively phytohemagglutinin stimulated T‐cell responses and particularly activation marker as well as CD40L/IL‐2 positive CD4^+^ T‐cell subsets were decreased as compared to serum from healthy contacts. Neither Staphylococcal Enterotoxin B‐mediated T‐cell stimulation nor phytohemagglutinin or Pam3CSK4 induced monocyte cytokines (i.e., Interleukin‐6, Interleukin‐8, Tumour Necrosis Factor‐α) were affected by the tuberculosis patients' serum samples. These results highlight the immunosuppressive influence of the tuberculosis serum milieu, which specifically reduced T‐cell responses to phytohemagglutinin, probably through impaired function of the accessory monocytes required for stimulation.

## Graphical abstract

TB serum selectively disrupts monocyte‐dependent PHA T cell activation and cytokines associated with proliferation while preserving SEB T cell and Pam3/PHA monocyte cytokine responses.

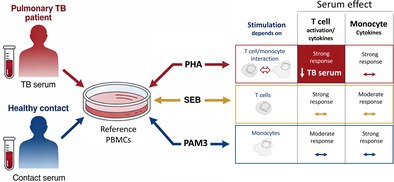

## 1 Introduction

Tuberculosis (TB) is a chronic infectious disease caused by species of the *Mycobacterium tuberculosis complex* (Mtb) in humans. The immune system plays a central role in controlling the pathogen after infection and can prevent the progression to TB disease in the majority of Mtb‐infected individuals. T cells and monocytes (as well as derived macrophages) are particularly essential for immune protection against Mtb infection. Progression to TB disease is associated with immunopathology, which in pulmonary TB leads to severe and often persistent damage to lung tissue. Chronic inflammatory changes are a typical feature of pulmonary immunopathology, and systemic inflammation is seen in a subgroup of pulmonary TB patients [1]. There is increasing evidence that blood immunopathology in TB affects T‐cell functions with a high similarity to chronic inflammatory diseases [2, 3]. A test used to assess the functionality of immune cells is the stimulation of T cells with mitogenic substances such as phytohemagglutinin (PHA). PHA is widely used today in clinical diagnostics for TB as a ‘positive control’ for interferon‐γ release assays (IGRAs).

PHA is a plant lectin that triggers the activation and proliferation of T cells in a monocyte‐dependent manner [4, 5, 6]. A reduced or completely absent response to PHA has been confirmed in TB patients by several studies [7, 8, 9]. This impaired response to in vitro PHA stimulation is seen in TB patients during the acute phase of the disease and decreases in the first weeks of anti‐mycobacterial therapy [9, 10]. We and others have demonstrated the important role of non‐cellular blood components—referred to as serum in this manuscript for simplicity, although previous studies have utilised both plasma and serum—on the impaired PHA response [11, 12]. The group led by J.J. Ellner was able to show as early as the last century that serum from TB patients could transfer the reduced PHA response to healthy immune cells (PBMCs) [11]. A modified version of this in vitro assay provided evidence that changes in the serum environment of TB patients are also responsible for pathological changes of monocytes [13, 14]. The exact mechanisms underlying serum‐induced impairment of the PHA response are still unknown. It is also unclear whether impaired T‐cell and/or monocyte functions or the interaction between the two immune cell subsets is influenced by changes in the serum environment in TB patients.

In order to answer these questions in the present study, we modified the previously performed in vitro serum assay to enable the concomitant analysis of TB serum milieu changes on cell specific activation and cytokine expression of monocytes and T cells. To achieve this, we compared the response of reference PBMCs to the mitogen PHA (a T‐cell stimulus dependent on monocyte support), the superantigen Staphylococcal Enterotoxin B (SEB; monocyte‐independent stimulus of T cells), and the synthetic TLR‐1/2 agonist Pam3CSK4 (Pam3; T‐cell‐independent stimulus of monocytes) in medium supplemented with TB or healthy contact serum samples. This approach allowed us to characterise the altered function of T cells and monocytes in terms of cytokine expression and activation, as well as the specificity of the TB serum milieu influence.

## 2 Material and Methods

### 2.1 Study Cohorts and Clinical Characterisation

Between February 2023 and October 2024, we recruited patients with TB (*n* = 30) and healthy household contacts of confirmed infectious TB patients (contacts; *n* = 28) in five hospitals in Kumasi and the surrounding Ashanti region (i.e., Kumasi South Government Hospital, Kwame Nkrumah University of Science and Technology (KNUST) Hospital, Atebubu‐Amantin Municipal Hospital, Sene West District Hospital, Agogo Presbyterian Hospital). Diagnosis of acute TB was made based on the patient's medical history, physical examination, chest X‐ray, sputum smear, sputum culture, and GeneXpert analysis, as previously described [14]. All TB patients were enrolled in the study before starting anti‐mycobacterial treatment. The contact persons had confirmed close contact with a TB index patient in the recent past. The medical examination of contacts did not show any symptoms of TB neither at enrollment nor during the study. The medical interview ruled out previous TB disease in contacts. A serum tube (Vacutainer; 5 mL) was taken from each TB patient/contact and processed according to the manufacturer's instructions. The serum was frozen in two tubes of 1 mL at −80°C until usage. The study groups were comparable in terms of age and sex and were selected as comparable pairs for the study design. The characteristics of the study groups are summarised in Table 1.

**TABLE 1:** Characteristics of study groups.

	TB	Contacts
Number of study cohort (*n*)	30	28
Mean age, years (range)	34 (17–85)	34 (18–82)
Male/female	12/18	12/16
HIV‐status	0/30	0/28
Diagnostic tests		
GeneXpert (pos/neg)	30/0	
Sputum culture (pos/neg)	29/1	
Sputum smear (pos/neg)	28/2	

The PBMCs used were obtained from heparinized whole blood (Vacutainer heparin tubes, BD) from a healthy donor who donated blood twice (70 mL each) within 4 weeks. The ethics committees of KNUST (CHRPE/AP/383/22) in Kumasi and the Medical Faculty of Heinrich Heine University Düsseldorf (ID: 2022‐2085_1) reviewed the study and raised no objections to its conduct.

### 2.2 PBMC Isolation and Stimulation in Medium Supplemented With TB Patient or Contact Serum Samples

PBMC isolation was performed directly after the blood take and density centrifugation (Ficoll, Biochrom, Berlin) was performed according to manufacturer's instructions. The cell culture was performed as described [13, 14, 15], however, serum (10% in culture medium) from TB patients and contacts was used in this study instead of EDTA blood plasma, as EDTA inhibits calcium influx‐based T‐cell receptor activation. In addition, PBMCs were used here to determine the response of both T cells and monocytes to the various stimuli (i.e., Staphylococcal Enterotoxin B (SEB; 1 μg/mL; Sigma‐Aldrich), the synthetic toll‐like receptor agonist Pam3CSK4 (Pam3; 100 ng/mL; Sigma‐Aldrich), and the mitogen lectin phytohemagglutinin (PHA; 2 μg/mL; Sigma‐Aldrich)) in the same approach. In two experiments, a total of 30 serum samples from TB patients and 28 serum samples from healthy household contact persons were used and cultured with PBMCs from the same donor and the respective stimuli. In summary, 2 × 10^5^ PBMCs were cultured in RPMI 1640 medium (Gibco) supplemented with L‐glutamine (1%; Sigma‐Aldrich) in 96‐well U‐bottom microtiter plates in a total volume of 150 μL for 16 h at 37°C and 5% CO_2_. Each sample contained serum (10%) from either a TB patient or a healthy household contact person. Separate approaches were performed for the measurement of activation markers on the plasma membrane and for the intracellular measurement of cytokines. For the intracellular analysis of cytokines, the Golgi inhibitor Brefeldin A (0.15 μL; concentration: 5 μg/mL; Sigma‐Aldrich) was added 3 h after the start of the experiment, which prevents the secretion of cytokines.

### 2.3 Staining and Measurement of Samples Using Multicolor Flow Cytometry

The samples were stored on ice immediately after culture and then processed to measure the activation markers and cytokines. To measure activation markers, the microtiter plates were centrifuged at 300 g and 4°C. The supernatant was then removed and the cells in the residual volume (approx. 20 μL/well) were stained with antibodies for 30 min on ice (protected from light). The following antibodies coupled to fluorescent dyes were used for this purpose (i.e., CD4 (BV510, clone: RPA‐T4; BioLegend), CD8 (FITC, clone: HIT8a; BioLegend), CD25 (BV421, clone: BC96; BioLegend), CD69 (BV605, clone: FN50; BioLegend), Viability dye (eFluor780; eBioscience)). The samples were then resuspended in PBS (4°C, 200 μL) and centrifuged (300 g, 4°C). After discarding the supernatant, this was repeated.

To determine the cytokines, the microtiter plates were centrifuged at 300 g and 4°C. The supernatant was then removed and the cells were stained in the residual volume (approx. 20 μL/well) with antibodies for 15 min on ice. The following antibodies were used for this purpose (i.e., CD4 (BV510, clone: RPA‐T4; BioLegend), CD8 (FITC, clone: HIT8a; BioLegend), CD11b (BV605, clone: ICRF44; BioLegend), anti‐HLA‐DR (PE‐CF594, clone: L243; BioLegend), Viability dye (eFluor780; eBioscience)).

The samples were then placed in PBS (4°C, 200 μL) and centrifuged (300 g, 4°C). After discarding the supernatant, this was repeated. The samples were then fixed and permeabilized (Fixation Buffer and Intracellular Staining permeabilization wash buffer, BioLegend) according to the manufacturer's instructions. The samples were then stained with the following antibodies for 30 min on ice (protected from light): IL‐2 (PerCP‐Cy5.5, clone: MQ1‐17H12; BioLegend), IFN‐γ (PE‐Cy7, clone: 74S.B3; BioLegend), TNF‐α (APC, clone: MAb11; BioLegend), CD40L (Pacific Blue, clone: 24–31; BioLegend), TNF‐α (AF700, clone: MAb11; BioLegend), IL‐8 (FITC, clone: BH0814; BioLegend), IL‐6 (PE‐Cy7, clone: MQ2‐13A5; BioLegend). The samples were then washed twice in permeabilization buffer (BioLegend) (see above). For measurement on the 4‐laser multicolor flow cytometer (LSR‐Fortessa, Becton Dickinson, USA), the samples for activation marker determination and intracellular cytokine analysis were each taken up in 100 μL PBS. The data was evaluated using FlowJo software (version 10, Becton Dickinson). The evaluation procedure is shown in Figures S1–S3. For qualitative analysis of cytokine expression pattern, analyses were performed as follows: CD4^+^ and CD8^+^ T cells expressing at least one of the 4 cytokines that is IL‐2, IFN‐γ, TNF‐α, and CD40L following stimulation with PHA and SEB were included in this analysis. Boolean gating was applied to determine the cytokine expression patterns of CD4^+^ and CD8^+^ T cells. Here, 15 different expression profiles comprising single, double, triple and four‐fold cytokine positive T cells were obtained and compared between different stimulations as well as between the study groups. To compensate for the significant differences in cytokine induction between PHA and SEB, the responses were normalised to 1 for each sample and the relative differences for each profile were presented.

CD11b and HLA‐DR were used to identify monocytes, as the classic monocyte markers CD14 and CD16 are subject to strong expression regulation in vitro, which is additionally influenced by various stimuli. It is therefore not possible to subdivide monocytes into classic and alternative subtypes in this assay.

### 2.4 Graphical Depiction and Statistics

GraphPad Prism v10 software (GraphPad Software, USA) was utilised for statistical analyses and graphical depictions. Nonparametric tests were employed because of the relatively small study group sizes. The Wilcoxon signed‐rank test was used to determine statistical significance between paired samples with and without stimulation. The Mann–Whitney *U* test was used to compare parameters between the study groups. A *p*‐value less than 0.05 was considered statistically significant.

## 3 Results

### 3.1 PHA and SEB Induce Different Activation and Cytokine Expression Patterns in T Cells

**FIGURE 1:**
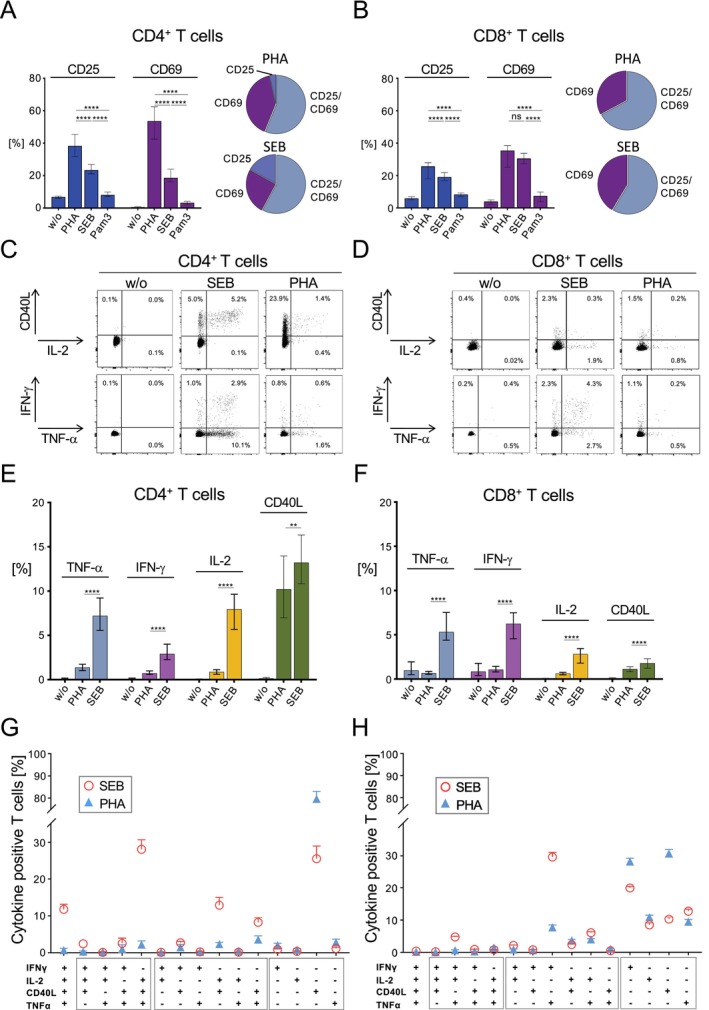
T‐cell activation and cytokine expression profiles after stimulation with PHA, SEB, and Pam3. PBMCs supplemented with serum samples from individuals of both study groups (*n* = 58) were stimulated for 16 h with PHA, SEB, and Pam3 or w/o. (A, B) Flow cytometry analysis of activation markers CD25 and CD69 expression on CD4^+^ (A) and CD8^+^ (B) T cells. (C to F) Candidate cytokine (CD40L, IL‐2, IFN‐γ and TNF‐α) expression in CD4^+^ (C, E) and CD8^+^ (D, F) T cells. The bar charts represent medians with error bars showing the 75th and 25th percentiles. (G, H) T cells positive for at least one cytokine after stimulation with PHA or SEB were classified for their expression pattern (i.e., positive for four, three, two, or one cytokine) and depicted as symbols (blue triangles for PHA; red circles for SEB) with standard error bars. Statistical significance between the different stimuli was assessed using the Wilcoxon signed‐rank test; asterisks indicate *p*‐values (**, *p* < 0.01; ****, *p* < 0.0001; ns, not significant).

Initially, we compared the expression of activation markers and cytokines in T cells after in vitro culture of PBMCs with the T‐cell specific stimulus SEB, the TLR1/2‐ligand Pam3, and the mitogen PHA. All samples supplemented with serum from TB patients and contacts (*n* = 58) were analysed together for this purpose. The gating procedures are depicted in Figures S1 and S2. T‐cell activation markers, CD25 and CD69, were strongly up‐regulated in CD4^+^ and CD8^+^ T cells after SEB or PHA stimulation, whereas Pam3 had only moderate effects on T‐cell activation (Figure 1A,B). CD25/CD69 double‐positive cells were the dominating phenotype in CD4^+^ and CD8^+^ T cells stimulated by SEB or PHA (Figure 1A,B). Next, intracellular measurement of cytokine expression was performed and compared between T cells after in vitro stimulation with the different stimuli. Representative dot plots of flow cytometry analyses are shown for CD4^+^ and CD8^+^ T cells in Figure 1C,D. PHA and SEB induced significant expression of the T‐cell cytokines TNF‐α (only in CD4^+^ T cells for PHA), IFN‐γ, IL‐2, and the CD40 ligand (CD40L) (Figure 1E,F). Pam3 is not shown because no significant cytokine induction was measurable. SEB was the stronger inducer of all cytokines in both T‐cell subsets as compared to PHA (Figure 1E,F). To compare cytokine expression patterns in response to SEB and PHA, we next analysed the relative contribution of T cells with distinct cytokine profiles to the overall T‐cell response (Figure 1G,H). In CD4^+^ T cells, PHA mainly induced CD40L single‐positive cells, but also co‐expression of CD40L with IL‐2 or TNF‐α was detected (Figure 1G). For SEB, also CD4^+^ T cells positive for all four cytokines were induced, and co‐expression of CD40L with other cytokines (e.g., with IL‐2/TNF‐α or with IL‐2) was more prevalent as compared to PHA (Figure 1G). IL‐2, IFN‐γ, and CD40L single‐positive T cells were most frequent in CD8^+^ T cells when stimulated with PHA (Figure 1H). Single positive T cells dominated also SEB stimulated CD8^+^ T cells, but here also IFN‐γ/TNF‐α double‐positive cells contributed to the response (Figure 1H). We concluded that T‐cell stimulation by SEB and PHA can be characterised and distinguished by determining activation markers and cytokine expression. Next, we analysed monocyte responses to the different stimuli in the same assay.

### 3.2 Monocyte Cytokine Expression Is Differentially Induced by Pam3, PHA, and SEB

**FIGURE 2:**
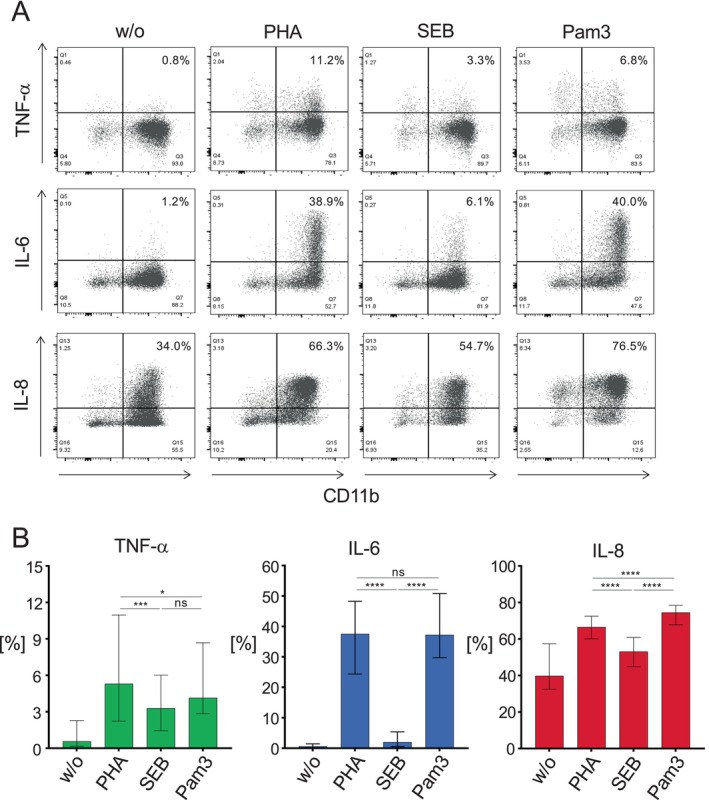
Monocyte cytokine expression profiles after stimulation with PHA, SEB, and Pam3. PBMCs supplemented with serum from both study groups (*n* = 58), and stimulated for 16 h with PHA, SEB, and Pam3 or w/o were analysed for monocyte cytokines. (A) Representative gating of flow cytometry dot plots showing the individual cytokines together with the monocyte marker CD11b. Each dot represents an individual cell, and quadrants are set based on unstimulated samples. Cytokine‐positive cells were quantified from the upper right quadrant. (B) Proportions of TNF‐α, IL‐6, and IL‐8 expressing cells under each stimulation condition are shown as bar charts, displaying medians with 25th and 75th percentiles. Statistical significance between stimulated and unstimulated samples was determined using the Wilcoxon signed‐rank test; asterisks indicate *p*‐values (*, *p* < 0.05; ***, *p* < 0.001; ****, *p* < 0.0001; ns, not significant).

In pilot studies, the cytokine response of monocytes to different stimuli was investigated, and three cytokines (i.e., TNF‐α, IL‐6, IL‐8) were selected for this study. TNF‐α, IL‐6, and IL‐8 showed distinct expression in monocytes in response to different stimuli (Figure 2A; Figure S3). Similar proportions of TNF‐α positive monocytes (about 4%) were induced by PHA, SEB and Pam3 (Figure 2B). In contrast, high proportions of IL‐6 positive monocytes (> 30%) were only induced by PHA and Pam3 while SEB had only moderate effects on IL‐6 expression (Figure 2B). In contrast to TNF‐α and IL‐6, IL‐8 was constitutively expressed by a significant proportion of monocytes in the absence of stimuli (Figure 2A,B). Higher IL‐8 expressing monocyte proportions were observed in response to all stimuli but PHA and Pam3 induced the highest level of IL‐8 in monocytes (Figure 2B). These results showed that intracellular measurement of cytokines can measure the response of monocytes to different stimuli. While SEB had only moderate effects on monocyte cytokine expression, Pam3 and PHA‐dependent activation induced marked and slightly different cytokine patterns.

### 3.3 TB Serum Samples Inhibit CD4 ^+^ and CD8 ^+^ T‐Cell Activation in Response to PHA but Not SEB Stimulation

**FIGURE 3:**
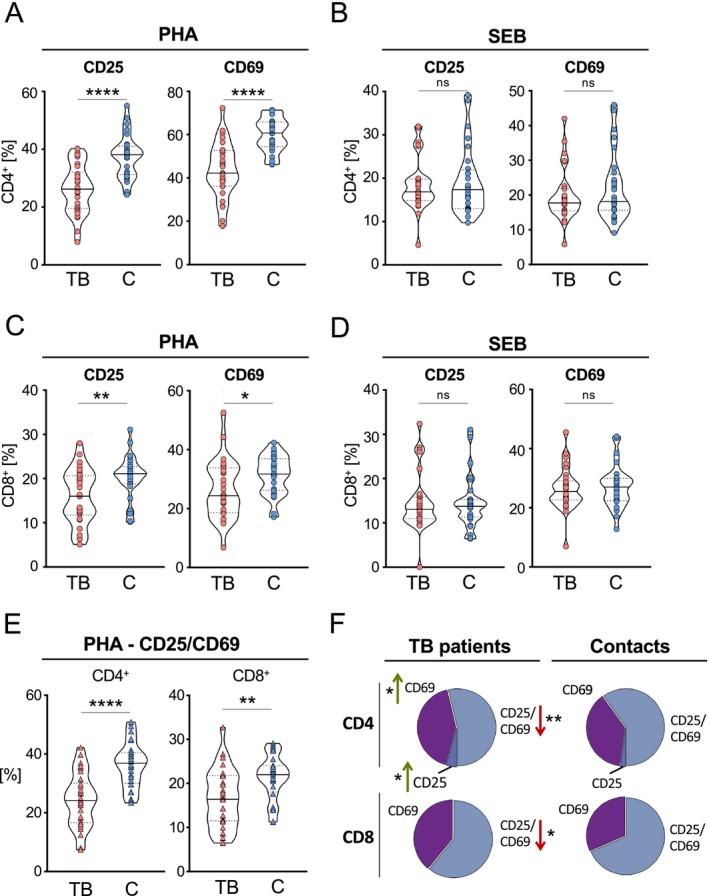
Inhibitory effects of TB serum samples on PHA‐induced T‐cell activation. Comparison of T cell activation marker expression in PBMCs supplemented with serum from TB patients (*n* = 30) or healthy contacts (*n* = 28), stimulated for 16 h with PHA (A, C, E, F) or SEB (B, D). (A–E) Activation marker expression on CD4^+^ (A, B, E) and CD8^+^ (C, D, E) T cells was quantified after subtraction of non‐stimulated control samples. Violin plots depict median proportions for individual samples (TB, red circles; contacts, blue circles) with 25th and 75th percentiles indicated as dotted lines. (F) Pie charts show proportions of single‐ and double‐positive activation marker–expressing T cells following PHA stimulation. Statistical differences between groups were assessed using the Mann–Whitney *U* test; asterisks denote significance levels (*, *p* < 0.05; **, *p* < 0.01; ****, *p* < 0.0001).

Cytokine expression differences in response to different stimuli observed in monocytes and T cells likely reflected the distinct pathways involved in T‐cell‐ (i.e., SEB), monocyte‐ (i.e., Pam3), and T‐cell/monocyte interaction‐dependent (i.e., PHA) stimulations. This enabled us to subsequently analyse the effects of serum from TB patients and healthy contact persons on these different immune cell subsets and pathways. Different serum samples caused considerable variability in the expression of the activation markers CD25 and CD69 after stimulation (Figure 3A–D). However, only PHA‐induced CD25 and CD69 positive T‐cell proportions were significantly reduced in the presence of serum samples from TB patients (Figure 3A,C). Both CD4^+^ and CD8^+^ T cells were affected, with the reduction in activated CD4^+^ T cells being greater on average (Figure 3A,C). In contrast, no significant differences were detected for SEB‐induced CD25/CD69 expression on CD4^+^ and CD8^+^ T cells between serum samples from different study groups (Figure 3B,D). The majority of PHA‐induced T cells expressed both, CD25 and CD69, and the proportions of double‐positive CD4^+^ and CD8^+^ T cells were also significantly reduced in the presence of TB serum (Figure 3E). To determine if activation marker single‐ or double‐positive T cells were equally affected, we determined the serum influence on the relative contribution of each subset among activated T cells (Figure 3F). Notably, the CD25/CD69 double‐positive were disproportionately reduced in both CD4^+^ and CD8^+^ T‐cell subsets and this was accompanied in CD4^+^ T cells by increased CD25 and CD69 single‐positive proportions in the presence of TB‐serum samples (Figure 3F). These results showed significant inhibitory effects of TB serum on PHA‐specific T‐cell response and particularly CD25/CD69 expressing CD4^+^ T cells were reduced in the presence of TB patients' serum samples.

### 3.4 TB Serum‐Mediated Inhibition of PHA‐Induced CD4 ^+^ T‐Cell Cytokine Expression Mainly Affected CD40L and IL‐2

**FIGURE 4:**
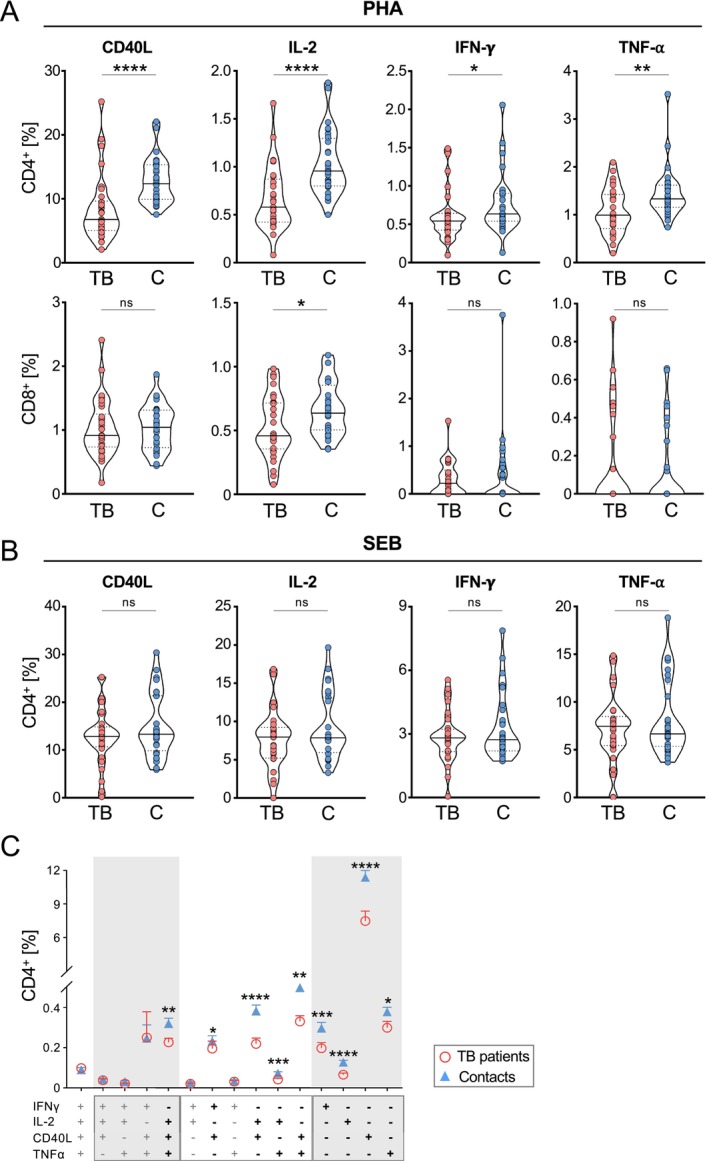
Inhibitory effects of TB serum samples on PHA‐induced T‐cell cytokine expression. Comparison of cytokine positive T cells between PBMCs supplemented with serum of TB patients or healthy contacts, and stimulated for 16 h with PHA (A, C) or SEB (B). (A, B) Cytokine‐positive CD4^+^ and CD8^+^ T cells were quantified after subtraction of non‐stimulated controls and are shown as violin plots, displaying median values for individual samples (TB, red circles; contacts, blue circles) with 25th and 75th percentiles indicated. (C) Cytokine expression profiles among PHA‐stimulated T cells positive for at least one cytokine were classified into cells expressing four, three, two, or one cytokine and are shown as symbol plots with medians and standard error bars. The legend indicates the presence of a cytokine with + and its absence with −. Statistical differences between groups were assessed using the Mann–Whitney *U* test; asterisks indicate significance (*, *p* < 0.05; **, *p* < 0.01; ****, *p* < 0.0001; ns, not significant).

Next, we compared serum effects on cytokine expression of T cells after stimulation. TB serum effects on PHA stimulation were particularly seen for CD4^+^ T cells (Figure 4A). CD40L, IL‐2, IFN‐γ, and TNF‐α expressing CD4^+^ positive T‐cell proportions were significantly reduced as compared to healthy contacts, but the median decrease of PHA‐induced CD4^+^ was strongest for CD40L and IL‐2 (Figure 4A). CD8^+^ T cells showed fewer changes overall in the presence of TB serum, and only IL‐2‐producing CD8^+^ T‐cell proportions were significantly lower in comparison with healthy contacts (Figure 4A). No significant differences were seen for SEB stimulated CD4^+^ or CD8^+^ T cells between serum samples from both study groups (Figure 4B; Figure S4). These results suggested PHA‐specific inhibitory effects of the TB serum on PHA stimulated T cells and possible differences on CD4^+^ T cells expressing different cytokines. To characterise these serum effects more precisely, we next compared CD4^+^ T cells with different cytokine profiles after PHA stimulation. Interestingly, T‐cell subgroups that produced only one or two cytokines were more strongly affected, and their proportions were lower in the presence of TB serum samples (Figure 4C). Multi‐cytokine‐positive T cells that expressed three or four cytokines were less affected, and among these, IFN‐γ‐positive T cells in particular did not differ between the study groups (Figure 4C). These results showed that CD40L/IL‐2‐positive CD4^+^ T cells were inhibited by the changes in the serum of TB patients in response to PHA.

### 3.5 Monocyte Cytokine Expression After Activation With PHA or Pam3 Was Not Affected by TB Serum Samples

**FIGURE 5:**
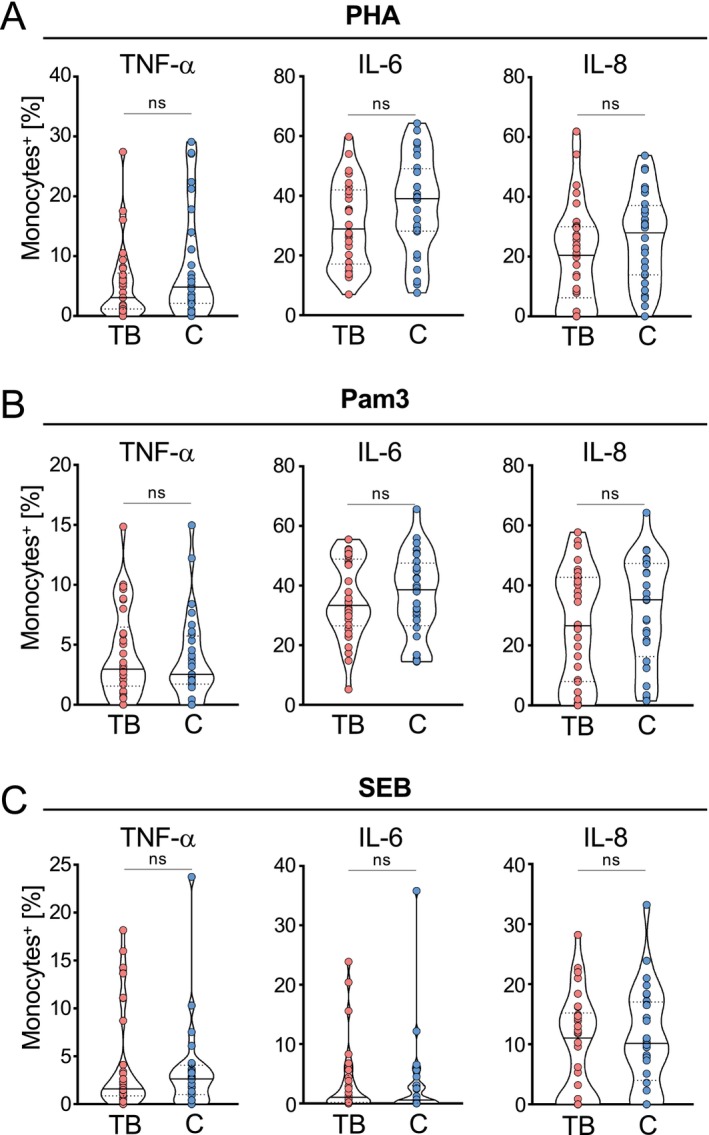
Similar monocyte cytokine expression between the study groups after stimulation with PHA, SEB, and Pam3. Comparison of TNF‐α, IL‐6, and IL‐8 positive monocytes between samples supplemented with serum of individuals from the study group of TB patients or healthy contacts in PBMCs stimulated for 16 h with PHA (A), Pam3 (B), or SEB (C). Calculated proportions of CD4^+^ T cells subtracted for non‐stimulated control sample are given. Violin symbol plots indicate median values for individual samples (TB patients, TB: Red circles; Contacts, C: Blue circles) with 75th and 25th percentiles as dotted lines. The Mann–Whitney *U*‐test was used to determine statistical significance between the study groups. ns: Not significant.

Finally, we analysed the monocyte response to the different stimuli in the presence of different serum samples. Monocyte phenotype and function were found to be strongly affected by changes in the serum milieu during TB [13, 14, 15]. However, PHA‐induced monocyte TNF‐α, IL‐6 and IL‐8 expression were only moderately and not significantly different in the presence of serum from TB patients or contact persons (Figure 5A). In addition, neither the monocyte‐specific stimulus Pam3, which induces strong expression particularly of IL‐6 and IL‐8, nor SEB showed TB serum‐dependent changes (Figure 5B). These results suggested that changes in monocyte function are not responsible for the impaired T‐cell response in TB, but rather that monocyte support of the T‐cell response to PHA is inhibited by TB serum.

## 4 Conclusions

T‐cell activation and cytokine expression, particularly CD40L, IL‐2 and TNF‐α, in response to PHA stimulation were significantly reduced in the presence of TB serum. Since neither the SEB nor Pam3 response of T cells or monocytes was affected by TB serum samples, we concluded that the interaction between T cells and monocytes required for PHA‐induced T cell responses is impaired by changes in the serum environment of TB patients. The fact that no significant effects of PHA‐induced monocyte cytokines (i.e., TNF‐α, IL‐6, and IL‐8) were observed suggests altered accessory cell‐dependent mechanisms, such as changes in costimulatory molecule expression, inhibitory ligand engagement, or contact‐dependent signalling. However, further research is needed to precisely characterise the underlying mechanisms.

## 5 Discussion

Immunopathological changes in the blood are a hallmark of tuberculosis, and the transmissibility of T‐cell dysfunction by serum samples of TB patients to healthy immune cells has been well‐documented [11]. The findings of this study indicated that the serum of TB patients exerted an inhibitory influence on the immune response exclusively to the lectin PHA under the stimuli employed. The in vitro activation of a T‐cell response against PHA is contingent upon the presence of cells from the monocyte/macrophage lineage [4, 6]. Conversely, SEB was shown to elicit T‐cell activation in the absence of other immune cells [16]. Activation and cytokine production of T cells following stimulation with SEB were not inhibited by serum samples from TB patients compared to healthy household contact persons. Therefore, a direct influence of TB serum on T cells during activation must be considered unlikely. TB serum effects on the function of monocytes, the necessary accessory cells in PHA stimulation, were another possible explanation. However, PHA induced monocyte cytokines such as IL‐6, IL‐8, and TNF‐α were not affected by TB serum samples, rendering a direct effect on monocyte responses improbable. This assumption was strengthened by the finding that the TLR‐1/2 agonist Pam3‐induced monocyte activation was not influenced by TB serum samples. These results suggested that the influence of TB serum particularly affects the interaction between T cells and monocytes, crucial for PHA stimulation without direct effects on T‐cell receptor or monocyte activation. Possible mechanisms involved include changes in costimulatory or coinhibitory receptor interactions, which increase the activation threshold of the T cell receptor, as well as the release of inhibitory cytokines.

PHA is known to bind to receptors on monocytes and T cells, and both types of binding can induce T‐cell activation [6]. Previous studies observed that soluble factors produced by monocytes also contribute to PHA‐dependent T‐cell activation [4]. However, we did not find any differences in the PHA‐induced monocyte cytokines measured in this study. This does not rule out an influence of the TB serum milieu on soluble factors secreted by monocytes, but does not prove it either. The influence of TB serum on the expression of receptors that are important for PHA‐induced monocyte/T‐cell interaction is also a conceivable explanation. Monocytes in particular show significant changes in phenotype in the presence of TB serum, and similar receptor expression differences are characteristic for monocytes from TB patients [13]. Global gene expression analyses identified an extensive group of genes that showed reduced expression on monocytes after contact with TB serum [14]. The functional implications of these changes are yet to be determined, but monocyte receptor expression changes are a possible explanation for the impaired interaction with T cells during PHA stimulation. Further studies are necessary to address this issue and exogenous modulation of candidates may elucidate mechanisms underlying the reduced PHA immune response.

There is evidence that the antigen‐specific immune response is also influenced by TB serum pathology. In this regard, TB patients with a low PHA response were also found to have a weaker response to *M. tuberculosis* proteins [11]. Interestingly, a similar connection is described for patients with chronic inflammatory diseases such as rheumatoid arthritis (RA). The impaired PHA response in patients with RA is well described [17, 18] and a correlation between reduced PHA and PPD responses has been demonstrated [7, 19]. In chronic inflammatory RA, the reduced PHA response was associated with high disease activity and normalised with successful treatment [20, 21]. In this context, it is interesting to note that TB serum also induces cytokine patterns in monocytes, which are typical of chronic inflammatory processes [14]. These similarities between TB and RA suggest similar mechanisms underlying the impaired PHA response and render a role for inflammatory changes likely. In accordance, a correlation between IL‐6 concentrations in the serum of TB patients and the reduced PHA response has been demonstrated [9]. Even though not all connections and underlying mechanisms have been clarified yet, the similarities between chronic inflammatory RA and TB suggest similar triggers and effects of the reduced PHA response in these different diseases.

The approach taken in this study has several limitations. For example, the short‐term incubation of reference PBMCs with serum‐enriched medium is a reductionist approach that reproduces the reduced PHA response in TB patients but does not take into account long‐term changes in T‐cell and monocyte function in TB pathology. Furthermore, it was not possible to investigate whether the expression of costimulatory or coinhibitory receptors on monocytes/T cells is influenced by TB serum samples, so the underlying mechanisms of impaired accessory monocyte function remain to be clarified. Finally, the strong phenotypic changes in monocytes in culture did not allow classification into described monocyte subpopulations. The established markers CD14/CD16 were significantly regulated in their expression and were therefore also not used to identify monocytes, but were replaced by CD11b in combination with HLA‐DR.

The results of this study support the hypothesis that the serum‐dependent reduced PHA response in TB patients is an indication of reduced interaction between monocytes and T cells. Although the exact mechanisms and molecules involved require further clarification, there is sufficient evidence for the immunopathological relevance of these changes. Novel immunomodulatory treatments (host‐directed therapies) should therefore include the immune response to PHA as a candidate biomarker, for example for therapy efficacy.

## Author Contributions

Conceptualization, E.M., J.S., R.O.P. and M.J.; methodology, J.F.A, H.S.A, M.M.V., D.M., A.Y., M.L., V.A.O., A.D.K, R.A., L.A.A, E.A. and D.O.O.; validation, J.F.A., D.M. E.A. and D.O.O.; formal analysis, J.F.A. and M.J.; investigation, J.F.A., E.A., R.O.P. and M.J.; resources, M.K.A., F.A.G., N.K.A.‐B., S.A., S.Z., E.M. and R.O.P.; writing – original draft preparation, J.F.A., J.S. and M.J.; writing – review and editing, J.F.A., J.S. and M.J.; supervision, E.A., D.O.O., E.M., R.O.P. and M.J.; project administration, R.O.P. and M.J.; funding acquisition, R.O.P. and M.J.

## Funding

This study was supported by the German Research Foundation (DFG, JA 1479/14‐1) and the graduate school molecules of infection (MOI)‐5 funded by the Jürgen Manchot Foundation. The funders had no role in study design, data collection, data analysis, decision to publish, or preparation of the manuscript.

## Conflicts of Interest

The authors declare no conflicts of interest.

## Supporting information


**Figure S1:** T‐cell activation Flow cytometry gating procedure and data processing. Duplet cells were excluded using a forward scatter height (FSC‐H) versus FSC area (FSC‐A) dot plot. Next, lymphocyte‐like cells were selected based on size (FSC‐A) and granularity (Side scatter area, SSC‐A). Viable lymphocytes were then selected. CD4^+^ and CD8^+^ T cells were subsequently gated, and the proportions of CD4^+^ and CD8^+^ cells expressing CD25 and CD69 with or without stimulation were determined.
**Figure S2:** T cell cytokine expression Flow cytometry gating procedure and data processing. Preliminary gating from Singlets to CD4^+^ and CD8^+^ is shown in Figure S1. Afterwards, CD4^+^ T cells (A) and CD8^+^ T cells (B) expressing TNF‐α, IFN‐γ, IL‐2 and CD40L, with or without stimulation were determined.
**Figure S3:** Monocyte cytokine expression Flow cytometry gating procedure and data processing. Duplet cells were excluded using a forward scatter height (FSC‐H) versus FSC area (FSC‐A) dot plot. Next, Monocyte‐like cells were selected based on size (FSC‐A) and granularity (Side scatter area, SSC‐A). Viable cells were then selected, followed by the HLA‐DR^+^ cells. Finally, proportions of CD11b^+^ cells (Monocytes) expressing candidate cytokines with or without stimulation were determined.
**Figure S4:** CD8^+^ T‐cell cytokine expression between the study groups after stimulation with SEB. Comparison of cytokine positive CD8^+^ T cells between samples supplemented with serum of individuals from the study group of TB patients or healthy contacts in PBMCs stimulated for 16 h with SEB. Calculated proportions CD8^+^ T cells subtracted for the respective non‐stimulated control sample are given. Violin symbol plots indicate median values for individual samples (TB patients, red circles; Contacts, blue circles) with 75th and 25th percentiles (dotted lines). The Mann–Whitney *U*‐test was used to determine statistical significance between the study groups. ns: not significant.

## Data Availability

The data that support the findings of this study are available from the corresponding author upon reasonable request.
