# Supplementary material for: Blood Immunopathology of Tuberculosis Patients Disrupts Monocyte‐Dependent T‐Cell Activation and Cytokine Expression
Source: Immunology. 2026 Mar 8;178(3):494–505. doi: 10.1111/imm.70131 (PMC13242981; doi:10.1111/imm.70131)
Supplement: Supplementary file 1 — Figure S1: T‐cell activation Flow cytometry gating procedure and data processing. Duplet cells were excluded using a forward scatter height (FSC‐H) versus FSC area (FSC‐A) dot plot. Next, lymphocyte‐like cells were selected based on size (FSC‐A) and granularity (Side scatter area, SSC‐A). Viable lymphocytes were then selected. CD4+ and CD8+ T cells were subsequently gated, and the proportions of CD4+ and CD8+ cells expressing CD25 and CD69 with or without stimulation were determined. Figure S2: T cell cytokine expression Flow cytometry gating procedure and data processing. Preliminary gating from Singlets to CD4+ and CD8+ is shown in Figure S1. Afterwards, CD4+ T cells (A) and CD8+ T cells (B) expressing TNF‐α, IFN‐γ, IL‐2 and CD40L, with or without stimulation were determined. Figure S3: Monocyte cytokine expression Flow cytometry gating procedure and data processing. Duplet cells were excluded using a forward scatter height (FSC‐H) versus FSC area (FSC‐A) dot plot. Next, Monocyte‐like cells were selected based on size (FSC‐A) and granularity (Side scatter area, SSC‐A). Viable cells were then selected, followed by the HLA‐DR+ cells. Finally, proportions of CD11b+ cells (Monocytes) expressing candidate cytokines with or without stimulation were determined. Figure S4: CD8+ T‐cell cytokine expression between the study groups after stimulation with SEB. Comparison of cytokine positive CD8+ T cells between samples supplemented with serum of individuals from the study group of TB patients or healthy contacts in PBMCs stimulated for 16 h with SEB. Calculated proportions CD8+ T cells subtracted for the respective non‐stimulated control sample are given. Violin symbol plots indicate median values for individual samples (TB patients, red circles; Contacts, blue circles) with 75th and 25th percentiles (dotted lines). The Mann–Whitney U‐test was used to determine statistical significance between the study groups. ns: not significant. [file IMM-178-494-s001.pdf]

# Figure S1

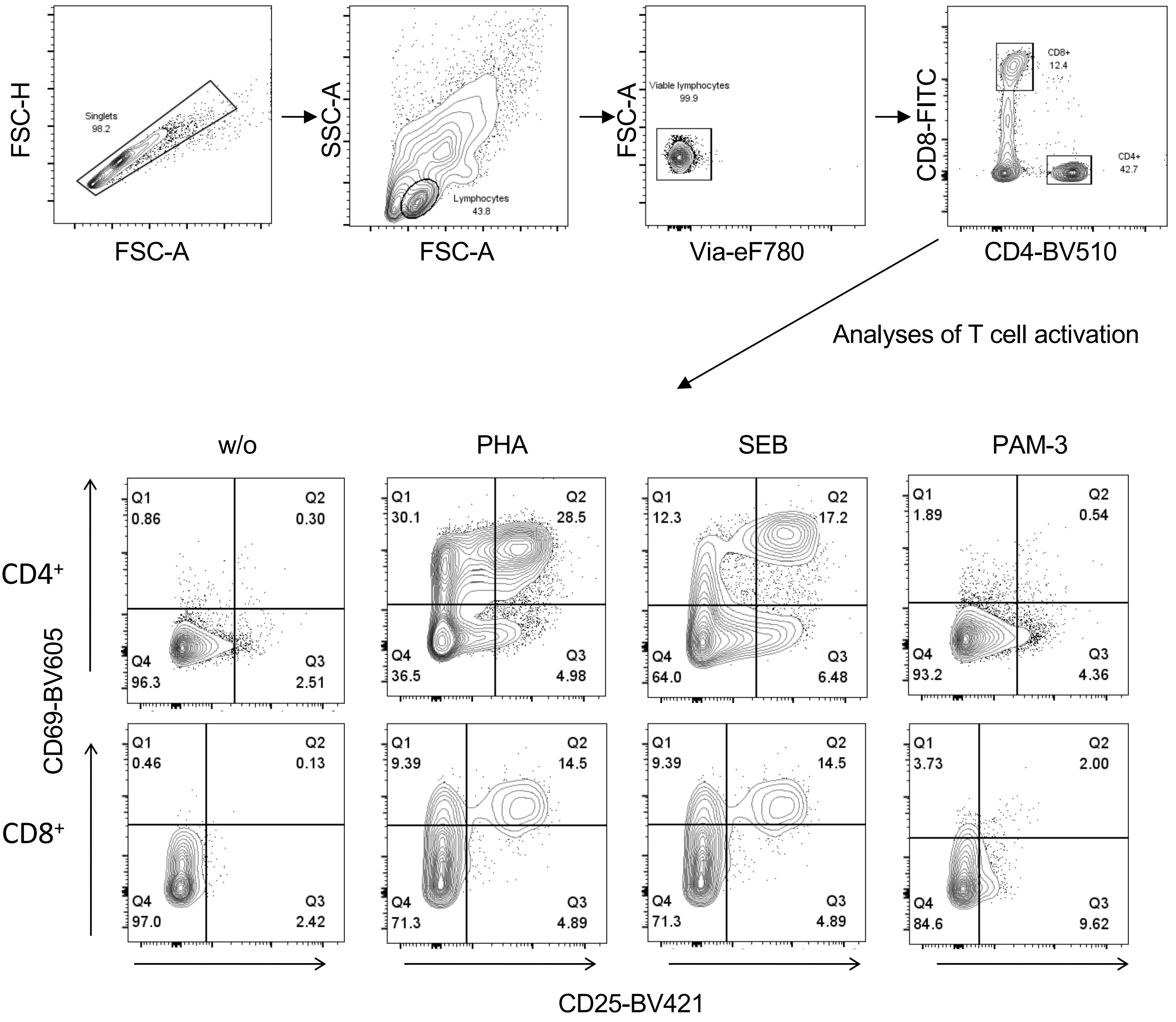


***T cell activation Flow cytometry gating procedure and data processing.*** Duplet cells were excluded using a forward scatter height (FSC-H) vs. FSC area (FSC-A) dot plot. Next, lymphocyte-like cells were selected based on size (FSC-A) and granularity (Side scatter area, SSC-A). Viable lymphocytes were then selected. $CD4^{+}$ and $CD8^{+}$ T- cells were subsequently gated, and the proportions of $CD4^{+}$ and $CD8^{+}$ cells expressing CD25 and CD69 with or without stimulation were determined.

# Figure S2

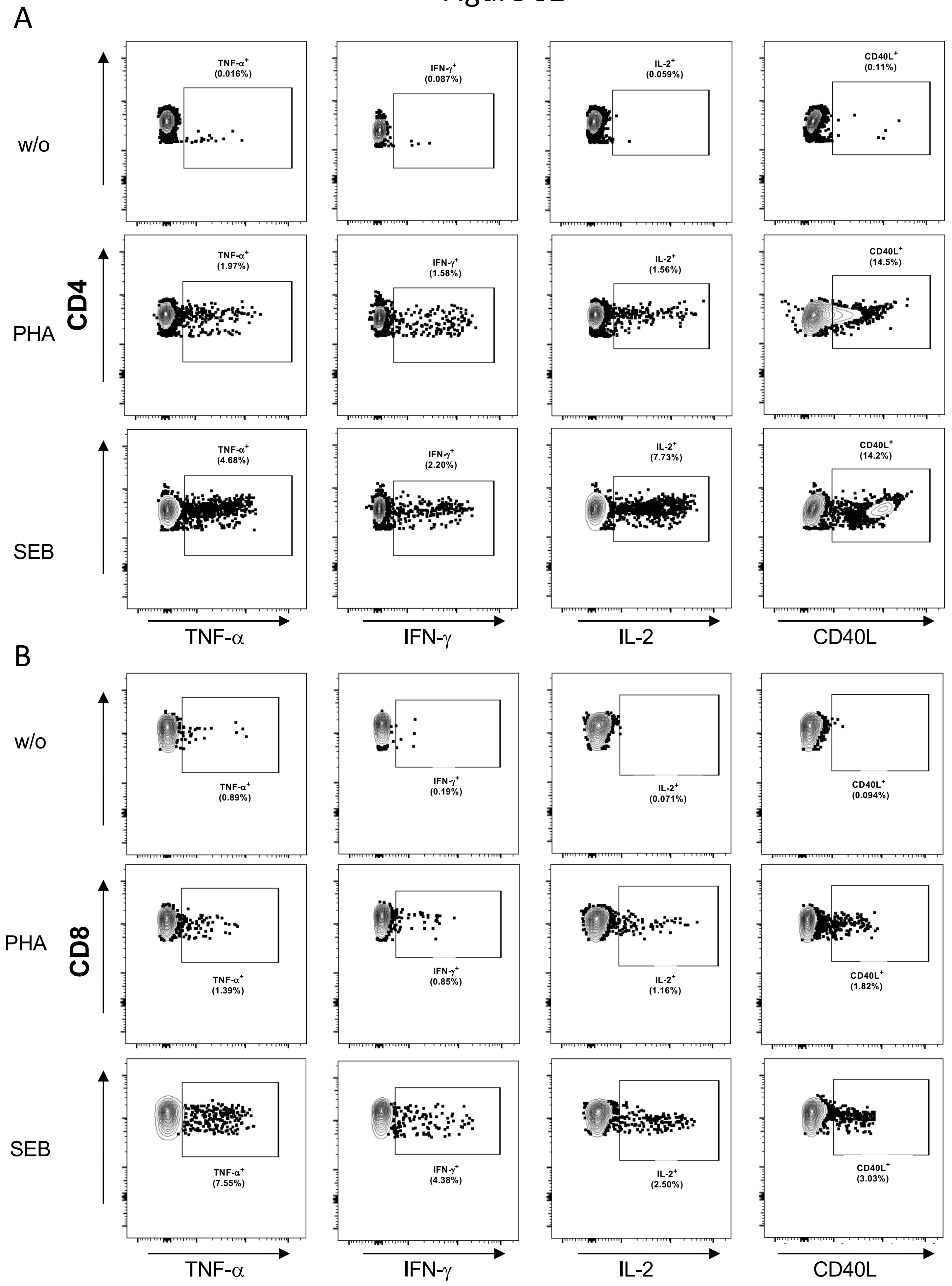


***T cell cytokine expression Flow cytometry gating procedure and data processing.*** Preliminary gating from Singlets to CD4+ and CD8+ is shown in Figure S1. Afterwards, CD4+ T- cells (A) and CD8+ T- cells (B) expressing TNF-α, IFN-γ, IL-2 and CD40L, with or without stimulation were determined.

# Figure S3

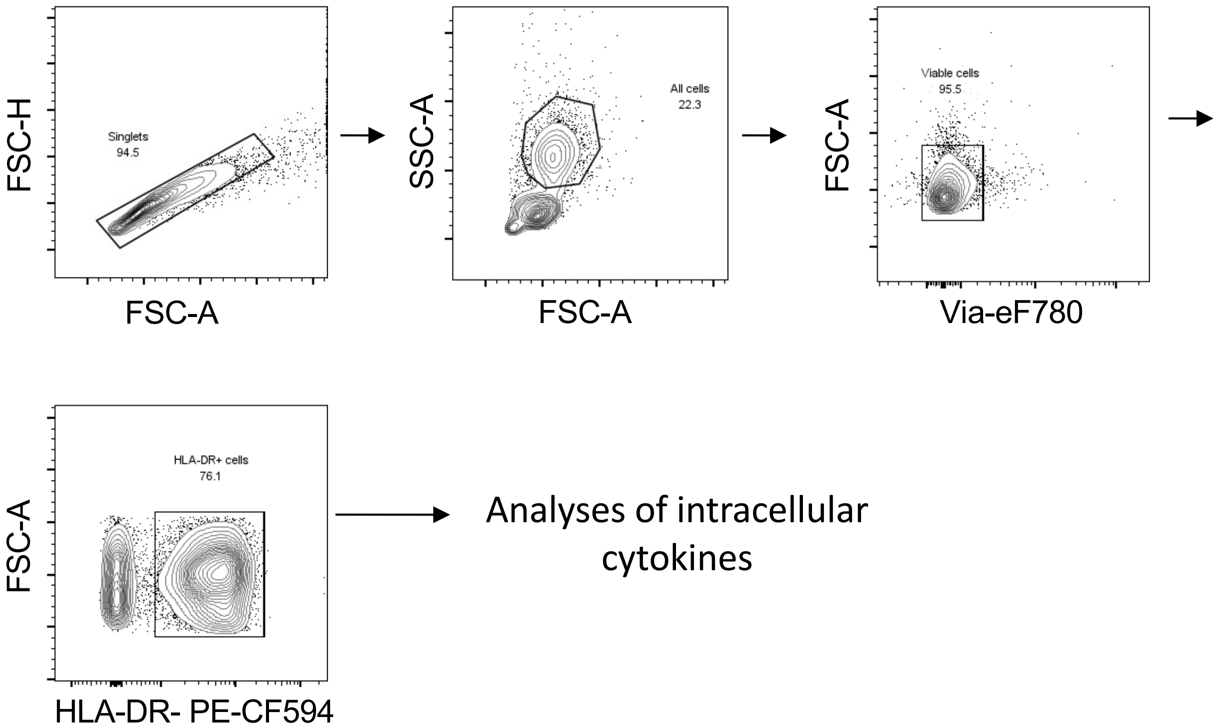


***Monocyte cytokine expression Flow cytometry gating procedure and data processing.*** Duplet cells were excluded using a forward scatter height (FSC-H) vs. FSC area (FSC-A) dot plot. Next, Monocyte-like cells were selected based on size (FSC-A) and granularity (Side scatter area, SSC-A). Viable cells were then selected, followed by the HLA-DR+ cells. Finally, proportions of CD11b+ cells (Monocytes) expressing candidate cytokines with or without stimulation were determined.

# Figure S4

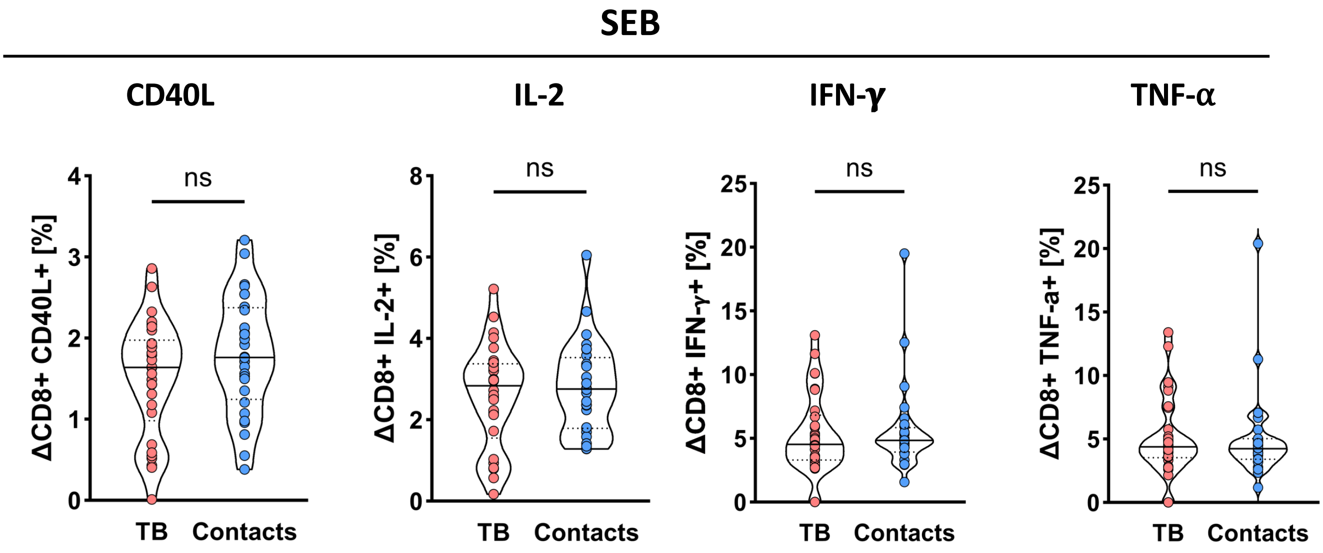


**CD8⁺ T-cell cytokine expression between the study groups after stimulation with SEB.** Comparison of cytokine positive CD8⁺ T-cells between samples supplemented with serum of individuals from the study group of TB patients or healthy contacts in PBMCs stimulated for 16h with SEB. Calculated proportions CD8+ T cells subtracted for the respective non-stimulated control sample are given. Violin symbol plots indicate median values for individual samples (TB patients, red circles; Contacts, blue circles) with 75th and 25th percentiles (dotted lines). The Mann-Whitney U-test was used to determine statistical significance between the study groups. ns indicates not significant.
